# Applying design to design: demonstrating how to create a human-centered design session interview guide for use with adolescents

**DOI:** 10.3389/fdgth.2025.1507517

**Published:** 2025-04-17

**Authors:** Macarena Kruger, Andrea B. Goldschmidt, Adrian Ortega, Abigail Wharton, Danielle A. N. Chapa, Erin R. Stalvey, Isabel R. Rooper, Katrina T. Obleada, Graham C. Miller, Andrea K. Graham

**Affiliations:** ^1^ Center for Behavioral Intervention Technologies, Northwestern University Feinberg School of Medicine, Chicago, IL, United States; ^2^ Department of Psychiatry & Behavioral Sciences, Northwestern University Feinberg School of Medicine, Chicago, IL, United States; ^3^ Department of Psychiatry, University of Pittsburgh School of Medicine, Pittsburgh, PA, United States; ^4^ Department of Preventive Medicine, Northwestern University Feinberg School of Medicine, Chicago, IL, United States; ^5^ Department of Medical Social Sciences, Northwestern University Feinberg School of Medicine, Chicago, IL, United States; ^6^ Department of Psychiatry and Behavioral Sciences (Child Psychology), Ann & Robert H. Lurie Children’s Hospital of Chicago, Northwestern University Feinberg School of Medicine, Chicago, IL, United States

**Keywords:** human-centered design (HCD), adolescents, digital mental health intervention, cognitive-behavior therapy, novel methodology, implementation science

## Abstract

Digital health interventions (DHIs) hold promise for improving the reach of mental health care for adolescents, particularly those from under-resourced communities who may face significant barriers to accessing in-person care. Yet, low engagement and uptake have challenged DHIs’ potency. Human-centered design (HCD) integrates end-users (i.e., future users of the DHI) into iterative design processes, thereby prioritizing their needs and preferences. Clinical scientists are increasingly embracing HCD, but often lack expertise in how to apply these methods in practice. We provide a template for creating a design session interview guide in a needs assessment, which is the first phase in our HCD process to design a DHI for dysregulated eating in adolescents. To create the guide, we first conducted a “needs assessment” within our team to identify important topic areas that required feedback from adolescents (“investigate”). We then consolidated these ideas into structured domains through a brainstorming process (“ideate”), which resulted in an initial draft of a design session guide (“prototype”). Next, we piloted the prototype with members of our team and a technology-savvy adolescent (“evaluate”) to refine it prior to administration with the target audience (“refine and develop”). Our internal needs assessment identified that we needed to learn adolescents’ preferences for technology (e.g., desired features), clinical content (e.g., areas for specialized support), delivery (e.g., coaching), and developmental relevance (e.g., focus on self-regulation). We organized these topics into six domains: dysregulated eating experiences and current help-seeking behaviors, major challenges that impact dysregulated eating, preferred intervention features and skills, preferences for coaching support, the potential role of sensors to assess activity behaviors, and preferred aesthetics and brand. We created relevant prompts within each domain, revised, and reordered them to elicit more comprehensive responses during administration. Next, we practiced administering the guide internally amongst our team, then with a non-participant adolescent volunteer. Using HCD, we created a semi-structured design session interview guide that will be administered in an upcoming needs assessment with adolescents and will continue to evolve as we learn from adolescents. This case example unpacks the process of creating and iterating a design session guide that could be applied across clinical domains.

## 1 Introduction

Digital health interventions (DHIs) hold promise for improving the reach of mental health care targeting adolescents, particularly those from under-resourced communities who may find cost and geographic location of in-person services prohibitive (1–3). However, DHIs have traditionally faced low engagement and limited uptake (4, 5). Too often, DHI tools are designed according to what researchers and industry experts think will elicit behavior change, rather than understanding what end-users (i.e., future users of the DHI) want, prefer, and need. This design problem is a principal reason DHIs often fail to engage end-users (6). In fact, adolescents have typically not been involved in DHIs’ design (7), despite being a tech-savvy population with high expectations for digital tools (1). Similarly, minoritized populations have often been excluded from design processes (8) and DHIs are “generally designed to be *one-size fits all*” [(6), p. 718], which limits their relevance to diverse populations.

Human-centered design (HCD) is a methodology that prioritizes the preferences of end-users by iteratively designing tools that meet their stated needs (9–12). One of the key principles of HCD lies in researchers recognizing and addressing their own knowledge gaps by engaging and partnering with end-users. This is particularly relevant when working with adolescents due to the generational differences between researchers and adolescents (13). HCD is a collaborative, cyclic, and iterative process, which often consists of six phases: (1) investigate, (2) ideate, (3) prototype, (4) evaluate, (5) refine and develop, and (6) validate. Numerous methodologies and techniques can be applied in each of these phases (4, 14, 15). See Table 1.

**Table 1:** HCD phases and techniques.

HCD phases	Concept	Techniques/examples
1. Investigate	Identify end-users’ needs, goals, and/or preferences	• Needs assessments: Inquire about end-users’ needs and preferences • Focus groups: Ask specific questions to a selected group to help design teams understand end-users’ thoughts/needs • Observations: Observe people or behaviors, without direct participation or interference • Market surveillance research: Understand key gaps in the market as well as similarities and differences between competitors • Expert consultation: Seek advice from professionals with expertise in the inquired areas
2. Ideate	Come up with different design ideas	• Brainstorming: Propose spontaneous ideas and identify potential design characteristics without following a specific order • Mind mapping: Write down a central theme and think of supporting and related ideas that expand the main theme • Storyboarding: Use text and visuals to communicate the proposed design and provide context
3. Prototype	Create variations of the product design	• Sketches: Create drafts of the product design (prototype) using photographs, illustrations, symbols, and/or words • Experience prototyping: Actively participate (e.g., through role-playing) as a team to capture potential real-life scenarios and experiences that end-users might have while engaging with the prototype
4. Evaluate	Iterate the prototypes with end-users to narrow down ideas	• Rapid iterative testing and evaluation (RITE): Identify early interface problems, fix them, and verify its efficacy through testing • Desirability testing: Gauge end-users’ first-impression responses to the prototype and make changes accordingly • Consensus within working groups: Compare the design team's perspectives on how to most effectively iterate/redesign specific components • Generative research: Invite end-users to participate in creative activities to obtain their insight on the prototype (e.g., manipulating design elements, creating collages, and drawing during design workshops)
5. Refine and develop	Finalize the design and develop the product (e.g., software)	• Shorten or expand the guide • Add specific items to refocus the guide towards the areas of greater interest • Iterate until a minim viable product (MVP) is obtained
6. Validate	Make the product available and/or test it in practice	• Single-arm open trials: Administer the prototype to a targeted sample of end-users • A/B testing and randomized controlled trials: Compare two versions of the prototype by randomly assigning different end-users to each version to determine which performs better in relation to a stated goal and/or expectations set • Qualitative methods: Conduct interviews, focus groups, and/or regular checkpoints to continue to understand fluctuations in end-users’ trust, desire, attitudes, and needs

Note: Other methods and techniques exist. This 6-phase process is just one model of the HCD process.

Applying HCD to the design of DHIs can improve engagement with DHIs (16), and in turn, enhance clinical impact (17). HCD can support behavior change processes by packaging clinical theories and principles in ways that are useful, usable, and applicable to real life scenarios. For instance, standard cognitive-behavioral therapy (CBT) interventions for dysregulated eating (i.e., maladaptive behaviors that cause distress/impairment and/or are associated with poor weight-related outcomes, such as loss of control eating, overeating, eating in the absence of hunger (18); are feasible, acceptable, and effective (19). As such, clinicians follow CBT principles to enhance individuals’ self-monitoring skills, to collaboratively create the personal formulation, and to introduce a pattern of regular eating [e.g., via food logs; (20)]. Importantly, HCD can inform how such a component intervention (e.g., food logs) could look and function within a DHI so that it is easy to find, easy to use, and provides the user (and potential others, such as coaches) with useful information to support behavior change. Furthermore, HCD can increase digital health equity by soliciting and centering the voices of individuals from diverse backgrounds (1). As such, HCD is increasingly being adopted for the design of DHIs.

However, clinical scientists often lack training and expertise in *how* to apply HCD methods to their research in practice (9, 21). For example, in preparing a design session interview, there may be uncertainty or lack of awareness of the types of questions that should be asked (i.e., we don't know what we don't know), the structure and order of the session, optimal ways to elicit key concepts from end-users, methods to iterate the interview guide over time to account for learnings and new ideas, and strategies to tailor design activities for particular populations. Indeed, there are additional considerations when designing for adolescents, such as parental involvement in both the design sessions and the product being designed (22), that can impact how HCD is applied.

Our research team is applying HCD to create a CBT-based DHI for dysregulated eating in adolescents. Our first research activity involves conducting design sessions using individual interviews with up to 20 end-users as part of a needs assessment (i.e., the “investigate” phase of the HCD process). Because most of our research team is new to HCD, we embarked on a systematic process to ensure group understanding of this methodology as we constructed our design session interview guide. The aim of this manuscript is to illustrate how we mirrored the phases of HCD to create an interview guide for design sessions with adolescents to learn more about their needs and preferences for a DHI that will incorporate CBT principles, as well as theories of self-regulation and executive functioning. Our process can provide a model for other clinical scientists developing design guides across different populations and clinical conditions/domains. Specifically, we provide a template to help researchers who are new to pursuing this work.

## 2 Materials and equipment

The team used Canva and Jamboard as design tools, particularly during the brainstorming process, including the “ideate” and “prototype” HCD phases of creating our design session guide. The internal administration of the design session guide with a non-participant adolescent volunteer was completed virtually using HIPAA-compliant Zoom.

## 3 Methods

The team was comprised of nine mental health professionals and clinical researchers at two academic medical centers. Five team members were clinical psychologists, one of whom was faculty with expertise in applying HCD to DHIs for adult eating disorders, two of whom were faculty with expertise in pediatric eating disorders, and two of whom were postdoctoral fellows with a training background in child psychology, health behavior, and DHIs. Additional team members included a research study coordinator, two research specialists, and a clinical psychology master's student, all of whom had experience conducting assessments and engaging the target population in clinical research.

Our process of creating the design session interview guide mirrored the phases of the HCD design process. First, we assessed our research team's needs by conducting a needs assessment within our team to identify the questions that would be important to answer in a needs assessment with adolescents (“investigate” phase). Even though our team was familiar with CBT and eating disorders in adolescents, our knowledge gaps revolved around what type of language we should use, what the intervention should look like, what components would be most critical in adapting CBT to a digital format, and how to increase adolescents’ engagement with the features. Additionally, because we are designing an app that aims to address self-regulation as a mechanism to improve eating behavior and intervention adherence, we consulted with a clinical scientist expert on strategies for improving executive functioning using behavioral interventions for eating-related behaviors/weight management. We also engaged in a “market surveillance” process to explore commercially available smartphone apps that target adolescents’ self-regulation (e.g., executive functioning skills), regardless of their focus on eating behaviors, to generate ideas on relevant app functionality to discuss with end-users. Then, we narrowed down these ideas into structured domains through a brainstorming process (“ideation”), which resulted in an initial draft of a design session guide (our “prototype”). Next, we iterated our design session guide prototype (“evaluate” phase), in which we revised the guide as a team asynchronously and then worked in small groups to make iterative updates. We subsequently conducted two rounds of usability testing to test its delivery. We first practiced administering the guide internally amongst our team. Then, we practiced administering the guide with a 12-year-old non-participant adolescent volunteer. Finally, based on the feedback obtained, we refined the prototype into our final design session interview guide (“refine and develop”). We will subsequently administer the guide in practice with diverse adolescent participants, to capture a broad range of perspectives, comparable to the “validate” phase of the HCD process.

## 4 Results

### 4.1 Investigating our needs & ideating a prototype

#### 4.1.1 Investigate

As a result of our internal needs assessment, we identified several knowledge gaps. We realized that we needed to learn adolescents’ preferences for the technology (e.g., type of device/platform that they typically use, desired features, how they would like to be represented in the app such as through their own avatar or pet), clinical content (e.g., areas for specialized support), support features (e.g., preferences for including a health coach), and developmental relevance (e.g., self-regulation skills, with a particular focus on executive functions).

Consultation with the expert on behavioral interventions targeting executive functioning helped us to gain insight around challenges that adolescents typically face due to the developmental process of brain maturation, particularly in neural regions related to self-regulation. The expert stressed the importance of providing adolescents with support around planning and self-monitoring skills. The expert also described how, in face-to-face treatment, they help adolescents identify planning skills they already use successfully in other domains (e.g., getting to school on time) and discuss strategies for applying these skills to eating. Consequently, we identified that our design session guide needed to include items that could help us better understand how a DHI could support adolescents’ planning processes and leverage skills they already use in other domains. We identified that we needed to prompt adolescents to share their preferences about using reminders, worksheets, and other supporting tools and preferred formats, as well as learn their constraints (e.g., times of the day that they are available/not at school).

By conducting the market surveillance research, we confirmed that there is a wide array of available smartphone apps that target adolescents’ general executive functions, in addition to specific food-related executive functioning skills. Commonly used techniques and skills were starting a habit tracker, completing word search quizzes, accessing a customizable toolbox, participating in a “slice setting” activity to break down the steps of a desired task to be accomplished, playing mini games that provide the opportunity for adolescents to reflect on the outcomes and how their emotions are triggered in real time, practicing mindful eating, receiving “fuel reminders” to eat and avoid skipping meals, and assessing their emotions through a “daily mood tracker” feature. The apps were identified by both searching commercially available apps in the App store as well as conducting a search on Google Scholar to explore whether there was research supporting their use, which would then inform the implementation of those tools in our DHI, copyright permitting. We also prioritized the inclusion of apps that targeted different domains of executive functioning (e.g., self-regulation, cognitive flexibility, inhibitory control, working memory). The lead author systematically documented different app features/categories, such as the “Description of the app,” “Target population,” “Implementation in the mental health field,” “Inclusion of self-regulation strategies,” “Format (e.g., word search, quizzes),” “Backed up by science,” “Cost,” and “Accessibility.” Overall, the consultation with an executive functioning expert and the market surveillance research enabled us to ask adolescents about their preferences for these types of activities and explore the possibility of eventually directing users to these existing tools or building similar tasks within our DHI. Importantly, we realized that we would not need to create executive functioning tools *de novo* because there are already “direct to market” games/practices available that can be readily implemented in our DHI.

#### 4.1.2 Ideate

The “investigate” phase illuminated knowledge gaps of the research team, which informed the structure of the design session interview guide such that it would optimize collection of information that we were lacking. Our initial brainstorming process yielded nine interview guide sections: (1) phone use, (2) current eating-related experiences and management strategies, (3) experiences with other health apps, (4) intervention skills, (5) features, (6) intervention designs, (7) coaching, (8) managing weight and dysregulated eating, and (9) using technology to manage weight and dysregulated eating. After engaging in multiple brainstorming sessions as a team, we narrowed these sections into six domains (See Table 2): (1) dysregulated eating experiences and current help-seeking behaviors (e.g., previous moments in which adolescents engaged in dysregulated eating and the solutions they have sought out); (2) major challenges that impact dysregulated eating (e.g., antecedents to these episodes, barriers to recovery); (3) preferred intervention features and skills (e.g., food log, planning tools); (4) preferences for coaching support (e.g., characteristics of the coach, modality of coaching such as phone or text messaging); (5) the potential role of sensors (e.g., to assess and intervene on real-time behaviors); and (6) preferred aesthetics and brand (e.g., interface design, DHI's name). We then created relevant question prompts within each domain. These domains and prompts resulted in an initial draft of our design session interview guide (“prototype”).

**Table 2:** Domains, subdomains, and sample questions: design session interview guide.

Domain number	Domains	Subdomains	Sample questions
1.	Dysregulated eating experiences and current help-seeking behaviors	• How do adolescents experience dysregulated eating? • How do they label/describe these episodes? • What skills have adolescents developed to avoid/bounce back from these episodes? • Do they use any techniques to manage these episodes, such as calling a friend or looking for information on the Internet that provides tips to regulate their eating behaviors?	• How long have you been struggling with eating in this way? • How much experience do you have in working on these behaviors?
2.	Major challenges that impact dysregulated eating	• What are common triggers for dysregulated eating episodes? • What barriers, such as social media content, do they perceive prevents them from engaging in more adaptive eating behaviors or addressing potential triggers?	• Are there any typical times, situations, or foods which make the experience of dysregulated eating more likely?
3.	Preferred intervention features and skills	• What intervention features would they find engaging to learn eating-related skills? • In what settings and contexts would they use an app to practice skills (e.g., at home, at night)? • To what extent do they want their parents involved in their intervention process?	• How do you think an app could help you reduce your episodes of dysregulated eating?
4.	Preferences for coaching support	• How and how often would they like to communicate with a coach? • What amount and type of data would adolescents be willing to share with their coach? • What identities are important to them in a coach (e.g., shared demographics, personal history)	• What information in the app, if any, should the coach have access to?
5.	Potential role of sensors	• What sensors/devices would adolescents be willing to wear? • Would they find sensors/devices helpful for improving their eating behaviors? • What are barriers to wearing/using sensors (e.g., school policies)?	• What are your thoughts about wearing a device that tracks your activity levels so the app could help you stay active at healthy levels?
6.	Preferred aesthetics and brand	• What colors do adolescents prefer in an app? • What images would they like to see in an app (e.g., illustrations vs. photographs)? • What name and images would they prefer in a logo?	• What would you want to call this app? • Tell me your reactions to [NAME] for the app.

Given our focus on augmenting a CBT-based DHI to target executive functioning, we added questions specifically around this topic within relevant sections. For example, in the section assessing “major challenges,” we included a question to target adolescents’ general executive functioning levels: “*Are there any typical times, situations, or foods which make this experience of having a dysregulated eating episode more likely?”* We also assessed self-regulation-focused skills, such as whether they have identified ways to track what they eat or self-monitor how their bodies react to food they eat (including the feelings that arise thereafter). Hence, we added questions such as, “*What feelings do you have when this happens? What happens after? Do your feelings change?”* Finally, in an effort to learn more about adolescents’ cognitive flexibility, we added a question about skill transfer/generalization (e.g., “*Is there anything that has helped you in the past?*”). Our goal is to garner insights on adolescents’ executive functioning levels so we can tailor our DHI features and design.

### 4.2 Evaluating the prototype

Once we had an initial draft created of our design session guide, we evaluated it to refine it for future administration in our study's needs assessment. First, we reviewed, revised, and reordered the design session guide prompts to elicit more comprehensive responses. For instance, we recognized the importance of building rapport to understand adolescents’ experiences and their past help-seeking behaviors prior to asking them about their input on a mobile-based solution for dysregulated eating. Doing so informed modifications to the guide, as we recognized the importance of orienting adolescents to the types of eating patterns about which we were soliciting information prior to evoking their own experiences. Accordingly, we started the design session guide by providing a simple definition of dysregulated eating episodes (loss of control eating and overeating) followed by asking adolescents to walk us through a recent “eating experience” and how they handled it.

We then practiced administering the design session interview guide. The first internal usability testing occurred with a team member (“simulated interviewee”), which yielded several areas upon which to iterate. We identified phrases that would be helpful to include in the guide to build trust and increase engagement, such as praising the participant for their effort responding to the questions and providing updates on timing (e.g., “*We are halfway through…. Are you okay to keep going?*”) to manage participants’ expectations throughout the session*.* The simulated interviewee also gave feedback to incorporate transitional phrases when going from one section to another (e.g., “*Now, we are going to switch gears and talk about how things look when you try to manage these experiences and the resources that would be most helpful*”). Additionally, there were takeaways regarding administration procedures, such as including a set of instructions (i.e., “to do” checklist) for the assessor to follow prior to, during, and after design sessions to ensure that the session runs smoothly (e.g., balancing notetaking while administering the session, screen-sharing visualizations during the session, starting and stopping the recording at appropriate times).

The second internal usability testing was conducted with an adolescent volunteer. This practice session yielded insights to improve the structure of the design session guide, as well as on how to better operationalize the concepts that we wanted to assess. The adolescent volunteer also provided feedback regarding the interview guide's length. Based on this feedback, we shortened the guide from 1 hour to 45 minutes to maintain future adolescents’ attention. In addition, we critically reviewed the guide and highlighted the prompts that seemed critical to ask. We then deleted those which seemed redundant, and demarcated those that could be asked as “follow ups” if time permitted.

As we revised the prompts, we continued to embrace removing our own assumptions for how the DHI “should” be packaged and instead centering end-user preferences in its design. For instance, we decided to provide fewer examples of the types of skills that the app could teach (e.g., meal planning, riding urges, mindful eating, regulation skills) to give adolescents the opportunity to come up with their own ideas of what might work for them. We acknowledged the need to ensure that our questions were open-ended without directing. Accordingly, we revised the prompts to get adolescents’ genuine feedback, being mindful about our own biases, by avoiding leading or suggestive questions that could influence participants’ responses at the expense of eliciting their own thoughts and preferences. Finally, the practice session with the adolescent volunteer underscored the importance of learning and using terms that end-users are familiar with, rather than using our own clinically-grounded terminology (e.g., for us, not referring to “dysregulated eating episodes” but using more familiar or acceptable terms like “eating experiences” to build rapport). To that end, we included a question to directly ask them: “*What do you call these experiences? Is there any particular “name” that you use?*”

### 4.3 Final refinements

After considering the insights from the internal usability testing administrations as well as the feedback from small group meetings among members of the research team, we reconvened as a full research team. We iteratively updated the guide by improving language and question flow, content, and procedural considerations within the six domains. We continued to tailor the prompts to make them developmentally and contextually appropriate for and appealing to adolescents. We also added questions addressing feasibility issues and practical barriers. For instance, we included questions about barriers that would prevent adolescents from engaging with an app. By doing so, we aim to learn if they have any technology access restrictions/fixed schedules and/or if there would be any other constraints that could interfere with them using an app for dysregulated eating. We also included a question about their perspectives on sharing a photo of their meals with the app, through which we expect to understand whether their parents/legal guardians may raise any privacy concerns and/or if adolescents will not be willing to take part in this activity on a regular basis.

We also finalized which research team members would lead the design session interviews with participants. We acknowledged the importance of the assessor having previous experience working with adolescents so that they can “speak their language.” Taking into consideration the feedback obtained from mirroring HCD phases, we revised the interview guide five times in total. This iterative process resulted in our finalized design session guide, which is now ready for administration (see Supplementary Material).

## 5 Discussion

As clinical scientists are increasingly encouraged to use HCD methods in the design of DHIs to improve uptake, this case example unpacks the process of creating and iterating a design session guide that could be applied across diverse clinical domains. Our approach to creating the guide modeled a HCD process (“applying *design* to *design”*), and therefore can serve as a template for future researchers new to design. Indeed, regardless of the clinical domain or developmental level of the population, this manuscript offers takeaway lessons on how to apply this methodology effectively.

For one, we embraced the importance of engaging in a comprehensive internal reflection process to have a clearer understanding of the types of questions that would be most beneficial to ask adolescents. Additionally, conducting internal administration testing before launching the design session guide with study participants served as a useful mechanism to obtain thorough and timely feedback. This strategy helps to maximize researchers’ and future participants’ time and the robustness of the data that could be elicited in a session, by screening the design session interview guide through a first “filter.” This is particularly relevant when interviewing adolescents, for whom it is critical to optimize time to minimize cognitive fatigue, boredom, and waning attention over time during a given task (23). These factors, which are characteristic of adolescents’ developmental stage, could alter the responses they provide (e.g., rushing to get through the remaining questions by providing simple, unelaborated, or non-informative answers). Overall, internal testing is valuable for research teams because it confirms that the design session guide is ready to be implemented in practice and prepares the research team for its administration.

Specific to the design session guide, our efforts yielded some key principles for other research groups to consider when preparing for interview-based needs assessments: (1) overall timing, (2) pacing, (3) “front loading” (i.e., prioritizing) key topics earlier in the session rather than later on, (4) rapport building, and (5) language. In terms of overall timing, it is important to be mindful of the session's length to prevent adolescents from reaching their maximum sustained attention capacity, which could differ across subpopulations. Regarding pacing, researchers should allocate time between sections depending on the item prompts’ levels of difficulty and adolescents’ engagement. Similarly, if certain sections or topics are of greater interest for researchers, those should be prioritized and potentially administered earlier than later (in case discussion of *a priori*tized topic takes longer than anticipated), whereas less time should be devoted to the less relevant ones. To facilitate rapport, we recommend praising the details that end-users share, inviting them to critique materials that are shown in the session (e.g., prototypes), emphasizing that there are no right or wrong answers, and requesting permission to move forward in sections. Lastly, researchers should use language that resonates with end-users (e.g., relevant and age-appropriate terminology) and reflect back the experiences that end-users share throughout the interview to confirm comprehension and demonstrate active listening. See Table 3 for additional internal considerations.

**Table 3:** Iterative questions for research teams to consider.

Guide considerations	Session considerations
Investigate: Identify what you do not know	
• Where are your team's knowledge gaps? • What do you need to learn from end-users? • What might you need to learn from others (e.g., outside experts)?	• Who on your team has conducted design sessions before? • What must you learn to confidently conduct these sessions? • Who can you consult with questions about conducting design sessions?
Ideate: Generate ideas and possible plans	
• Of the range of topics identified, which topics *must* you ask about? • Which topics would be useful to ask about, if time allows? • What domains might your topics group into?	• What has worked well or poorly for you when conducting past semi-structured interviews? • Who on your team will conduct the design sessions? • How might team members’ identities influence end-users’ responses?
Prototype: Draft your guide and session plan	
• What specific questions and prompts are needed to elicit information from end-users? • What other questions and prompts would be relevant to include to inform your product's design?	• What session length is appropriate for your end-users? • How should the interviewer(s) introduce themselves and build rapport throughout the session?
Evaluate & Refine: Iterate and revise your guide and session plan	
• Will these questions elicit the information you want to elicit? • Are you missing anything? • Does the order of your questions make sense? • Is your phrasing developmentally and culturally relevant?	• Is your communication style accessible? • How will you address power dynamics in the interview? • How can you help end-users feel safe and respected sharing their opinions? • How will the interviewer prepare (e.g., through practice administrations)?
Validate: Deliver your guide and session plan with end-users	
• Are your questions helping you learn what you needed to learn? • If not, how can you revise them? • What new ideas are arising that you want to ask more about?	• How is the interview pacing? • (As needed) how can you restructure the guide to maintain momentum? • Are sensitive topics discussed at a good time (e.g., after building trust at certain point of the session)?

Note: Researchers can consult these example questions as they develop their design session guide. As saturation emerges (e.g., when the team feels consensus has been reached), they can return to these questions to iteratively update their guide and refocus on the remaining unknowns.

Inherent to HCD is iteration based on end-users’ feedback provided throughout the design process (24). The interview guide we created will change over time as we progressively learn from a broad array of end-users to garner diverse perspectives and preferences (11) and reach saturation. Hence, the prompts will continue to evolve as we learn from adolescents’ needs and experiences, which will make it necessary to refine, update, or eliminate certain questions or domains as saturation in responses is reached (2).

Additionally, the flow of prompts within our current design session guide mirrors the HCD process, which starts by broadly inquiring about end-users’ needs and preferences, and becomes progressively specific as researchers start to inquire about a solution (e.g., a DHI). Our current guide is purposefully broad in focus because our internal team identified significant knowledge gaps and generational differences (e.g., what adults find helpful, engaging, and/or funny is not necessarily the same for adolescents) that need to be addressed before we can add questions to our guide about specific visuals, content, and features. Once we begin learning from end-users, we will progressively narrow our questions and refocus on domains that require further exploration. Overall, the balance between broad learnings and specific responses to particular design elements depends both on the types of research questions researchers seek to answer and where they are in the research process.

Further, we encourage researchers to be mindful about design equity constraints and the ethical burden that starting too broad could pose for end-users from traditionally underserved populations. Even though valuable insights can result from encouraging end-users to think about desired DHI features that are not necessarily rooted in reality, so called “blue sky thinking” can be a “luxury practice that marginalizes those who have endured life with systemic disadvantage and resource scarcity” [(25), p. 12]. It is imperative for researchers to acknowledge the challenges that traditionally underrepresented populations face related to visualizing equitable design solutions. We should strive for design equity by asking end-users about potential DHI features that encourage their creativity but that, to a certain extent, are also feasible across populations. That way, researchers can minimize undesired consequences related to design inequity.

Indeed, although we did not recruit participants here to create our design session guide (given the methodological nature of this paper), the next phase of our study involves capturing a broad array of perspectives to design our DHI. We are developing a recruitment plan to solicit a sample of adolescents from diverse backgrounds who have lived experience of dysregulated eating. We will be distributing flyers in community centers (e.g., high schools, libraries, parks), reaching out to “community champions,” and launching recruitment posts on online social media platforms. Augmenting local recruitment strategies with online platforms enables researchers to recruit individuals across the country, which facilitates capturing a wider (and more representative) range of perspectives. We also are initiating a Teen Advisory Board to engage a group of adolescent “consultants,” with or without lived experience of dysregulated eating, to provide ongoing feedback on general aspects of the intervention such as language and visuals (rather than aspects specific to eating). Partnering with adolescent advisors is another strategy we are using to capture diverse perspectives.

Finally, we recommend researchers who work with minors to pay special attention to measures regarding confidentiality, privacy, and research ethics. Neelakantan and colleagues (2023) suggest “assessing caregiver consent requirements and obtaining adolescent views on study documents and measures” (p. 1405). Accordingly, when we administer the design session guide in practice with adolescent participants, we will require a parent or legal guardian to be present during the videocall with their camera on to provide consent. Concurrently, the adolescent will assent to participate in the study, after the assessor confirms that the adolescent understands what their participation in the study entails. Once consent/assent is obtained, the parent/legal guardian will be asked to leave the call with the purpose of ensuring privacy and maintaining the adolescent's confidentiality (28). Given that sensitive topics may cause emotional burden on adolescents (26), we encourage researchers to be as flexible as possible both with the questions being asked (e.g., keeping them open-ended) and with adolescents’ requests (e.g., skipping a question/whole section if needed). We also encourage researchers to disseminate ways that they mitigated these issues in their own research.

## 6 Conclusions

Clinical scientists and research teams that aim to create a needs assessments using HCD may not need to be as highly structured as we were in this process. However, we found our process to be valuable in aligning and preparing our team, who began this collaboration with diverse prior experiences applying HCD methods. Research teams may also benefit from other methods besides individual interviews for their needs assessment (e.g., focus groups, observations, A/B testing, affinity diagramming (14, 27), which should be chosen based on their research question, data needs, and administration constraints. Our goal with this manuscript was to illustrate a case example for how research teams can begin engaging with HCD methodology when designing DHIs.

## Data Availability

The original contributions presented in the study are included in the article/Supplementary Material, further inquiries can be directed to the corresponding author.
