# Supplementary material for: Applying design to design: demonstrating how to create a human-centered design session interview guide for use with adolescents
Source: Front Digit Health. 2025 Apr 17;7:1507517. doi: 10.3389/fdgth.2025.1507517 (PMC12043636; doi:10.3389/fdgth.2025.1507517)
Supplement: Supplementary file 1 [file Datasheet1.pdf]

**Supplementary Material. Design Session Guide**

**Instructions:**

Hello, thank you for being here today! This session will take approximately 45 minutes. Let me tell you more about how you can help today. We are designing a mobile app to help teenagers manage their eating and feelings related to their body. Your thoughts and experiences are very important to us because we want to design a tool that meets your needs.

We will ask you different questions and may present different ideas for the design of the app. We are not testing you, and there is no such thing as a wrong answer. We won't share your answers with anyone unless you want us to, or unless you are engaging in behaviors that might be harmful to you. Your responses will help us understand how we can improve the design and make it most engaging and relevant for teenagers like you.

[As needed] You will also see some of the initial design features that we have put together. We may ask you to talk out loud your thoughts so that you can respond with whatever is going through your mind. There is no need to think a lot about your answers. We are interested in your first impressions.

To support this project, this session will be video and audio recorded.

Do you have any questions before we begin?

[Note for the researcher] Remember to praise and reinforce participants' responses. For instance, you could say, "Oh, this is so helpful" "You are giving such great information" "I appreciate all that you are sharing… we'll get there in a little bit… Can I ask you this other question to better understand your experience?"

Okay, let's begin!

**Part 1: Loss of control eating/overeating experiences & help-seeking behaviors**
[Introductory – 5 minutes]

We will start by talking about the times in which you have eaten too much or may have had a feeling of loss of control while eating. Different teens describe loss of control when it comes to eating, as times when they've had a difficult time controlling what or how much they were eating or feeling driven to keep going. We want to get to know you and hear more about your experiences.

[ ] Think of a recent experience you had. Walk me through what happened.
[ ] What do you call these experiences, is there any particular "name" that you use?
[ ] How long have you been struggling with eating in this way?

[ ] *How much experience do you have in working on these behaviors? [If any] Tell me more about that. Where did you seek information? Have you used an app before? [If so] What do the current apps available do well/poorly?*

**Part 2: Major challenges**
[10 minutes]

I will now ask you about the challenges these experiences may be causing you, which will help us come up with ways to help people using our app.

[ ] How do these experiences impact your day-to-day life…have these experiences created problems for you?
[ ] What things may have prompted this?
[ ] Do you think being alone/being with others impacted this experience?
[ ] Are there any typical times, situations, or foods which make this experience more likely?

[ ] *What feelings do you have when this happens? What happens after? Do your feelings change?*

**Part 3: Intervention skills & features**
[10 minutes]

Now, we are going to switch gears and talk about how things look like when you try to manage these experiences and the resources that would be most helpful.

[ ] What are some things that you think would help you in the experience that you just described – is there anything that has helped you in the past?
[ ] How do you think an app could help?
[ ] What features would you expect to see in an app designed to support these experiences?
[ ] Do social situations impact your eating? What about family? What about social media?
[ ] Information can come from lots of source – like our friends and family, the internet, and even social media…what makes you feel like you can trust a source of information?

[ ] What would make you want to use an app versus another mental health resource?
[ ] How would you know something has improved or changed for the better? What would any change look like for you?
[ ] Is there anything specific you would want to learn about experiences like yours?
[ ] What would make you stop using the app? What would help you keep using it?

[ ] *What are some things you have tried to make different/healthier decisions? Is there anything that motivates you to work on these experiences? Is there anything that gets in the way? How can people best support you in challenging situations? What would get in your way in terms of engaging with an app? If you knew one of your friends was engaging in something harmful for them, how would you convince them to try something different?*

[ ] *We have some ideas about potential skills the app could teach you: meal planning, riding urges, mindful eating, regulation skills [elaborate as needed] – what are your thoughts? What would you like? What would you not like?*

Thank you so much for answering all these questions! We're about halfway through the interview.

**Part 4: Coaching**
[5 minutes]

As part of this app, you will be assigned a coach, who will provide support, guidance, and keep you on track with your goals.

[ ] Who would you want to be your coach? This could be someone around your age, an adult in recovery from eating challenges, or a clinical expert like a medical professional.
[ ] How would you want to communicate with your coach? Would you prefer texts, phone calls, or video chats?
[ ] How often would you like to get check-in or feedback from a coach?

[ ] *What information in the app, if any, should the coach have access to?*

[ ] *What information would you like to know about the coach? What information should the coach know about you?*

**Part 5: Sensors**
[7 minutes]

We also want to know your thoughts about wearing different devices that could help the app learn more about your health patterns and help you stay on track with your goals.

[ ] For example, we want to help teenagers engage in healthy levels of exercise or physical activity. What are your thoughts about wearing a device that tracks your activity levels so the app could help stay active at healthy levels? What concerns might you have about this kind of device?

[ ] Some devices are better than others for monitoring your physical activity. For example, this is an ActiGraph device that we use in research [show picture/device]. It fits on your non-dominant wrist. It's about [explain dimensions]. What are your thoughts about wearing this device? How could wearing this be helpful for you? Would it matter to you whether you could be able to see your physical activity patterns on the app like being able to see how much you have exercised? Is this something you would be willing to wear every day all day (24 hours)? What would get in the way of wearing this device?

[ ] *Additional prompts: You might have also seen or worn other devices to track your exercise like a FitBit or a Garmin. Here's some examples of what those look like [show pictures]. These also fit on your wrist. What are your thoughts about wearing these devices? Would you rather wear one of these compared to the research device we showed you before?*

[ ] Research tells us that teenagers' eating habits change depending on the location they are in. We want to help teenagers understand how their own eating and activity patterns might change across locations so we can help them make healthy decisions no matter where they are. We could do this by having the app monitor your location and having you answer brief questions about your eating and activity habits in different locations. What are your thoughts about the app tracking your location and sending you some questions to answer about your eating? Would you find it helpful if you could understand how your eating behaviors might change depending on your location?

[ ] *Additional prompts: We can also track your location using a GPS device that looks like this [insert picture]. How would you feel about carrying this device with you to track your location? Would you prefer the GPS or the app for tracking your location?*

[ ] There are also necklaces that exist for detecting your eating patterns. These necklaces can help you and the app understand your eating habits without you having to directly tell us or answer questions about. It's because the necklace can sense how fast you're eating and when you're taking bites. A device like this could be useful for helping you in the moment with your eating. What thoughts do you have about wearing one of these necklaces if it could help the app learn about your eating and support you in the moment? How could a necklace like this be helpful for you? Let me show you what this looks like. What would get in the way of wearing this device?

[ ] Some people use food logs to record what they ate. These food logs can take a lot of time to complete. Let's say instead of having you complete a food log of what you eat, we asked you to take a picture of your meal and snacks before and after eating. What are your thoughts about sharing a photo of your meals with the app?

**Part 6: Defining the app name**

[ ] What would you want to call this app?
[ ] We are thinking about the name "Vibe" [It stands for "virtual intervention for binge eating"]. What do you think about this name? Nothing you say will personally offend any of us. We really

want to know what you think about the name that we had in mind and learn about any other suggestions you might have.
